# Fault Diagnosis of Motor Bearing Transmission System Based on Acoustic Feature Fusion

**DOI:** 10.3390/s26175671

**Published:** 2026-09-07

**Authors:** Long Ma, Yan Zhang, Zhongqiu Wang, Bohao Niu

**Affiliations:** 1 CCTEG Shenyang Research Institute, Shenyang 113122, China; malongccteg@163.com (L.M.); zhangyanccteg@sina.com (Y.Z.); 2 State Key Laboratory of Coal Mine Disaster Prevention and Control, Xuzhou 221116, China; 3 School of Mechanical and Electrical Engineering, China University of Mining and Technology, Xuzhou 221116, China; ts24050045a31@cumt.edu.cn

**Keywords:** feature fusion, deep learning, fault diagnosis, transfer learning, time-series imaging, convolutional neural network

## Abstract

Bearings are crucial components in motor bearing transmission systems because they reduce friction and support loads. Therefore, bearing fault diagnosis is particularly important. This paper proposes a fault diagnosis method for motor bearing transmission systems based on acoustic signals and acoustic feature fusion. The complete acoustic signal is segmented, and seven time-series imaging methods, including Gramian Angular Difference Field (GADF) and Gramian Angular Summation Field (GASF), are used to convert one-dimensional signals into two-dimensional feature maps. The generated images are then input into a RegNet-based transfer learning network. According to the single-feature training results, the feature map datasets ranking in the top two, three, and four are selected for feature fusion to construct new datasets. The results obtained under the present experimental setup indicate that acoustic feature fusion can improve the diagnostic performance compared with using a single feature map dataset. After comprehensive comparison, the dataset generated by summing two feature maps, namely STFT and Mel spectrogram, is selected as the final input dataset in this study. The current work focuses on a fixed operating condition, and further validation under different speeds, loads, sensor positions, background noise levels, bearing models, and defect severities will be conducted in future work.

## 1. Introduction

The transmission system, a critical component of the mechanical system, plays a key role in transferring power from one location to another or altering the form and characteristics of power [1]. The motor bearing transmission system is an important part of the motor system. In this system, the motor, bearings, and related transmission components collaborate to convert electrical energy into rotational mechanical energy, which powers various equipment and machinery. Bearings, essential components within this system, have crucial functions that include reducing friction and supporting loads [2]. Therefore, fault diagnosis of bearings is essential.

In both academia and industry, bearing fault diagnosis stands as a fundamental research subject [3,4,5,6]. Scholars in academia have extensively employed various vibration diagnosis methods in their research endeavors. These methods include envelope analysis [7], wavelet transform [8], empirical mode decomposition [9], morphological wavelet slices [10], sparsogram [11], wavelet denoising [12], envelope order tracking analysis [13], cepstrum calculation [14], as well as tachometer-less synchronously averaged envelope feature extraction technique [15], etc.

In some circumstances, it may not be possible to fulfill the contact acquisition requirements of a vibration sensor, thereby rendering it impractical to place the sensor directly on the object under study. In such instances, exploring non-contact measurement methods becomes essential to procure the necessary signals for experimentation [16]. Compared with using vibration sensors to collect traditional narrowband vibration signals, using acoustic sensors to collect wider-bandwidth sound signals is a feasible method [17]. The application of sound signals for diagnosing motor bearing faults is not widespread; few scholars are currently engaged in research efforts within this domain [16,18]. Consequently, the relatively limited exploration of utilizing sound signals for motor bearing fault diagnosis suggests promising research opportunities in this area. Acoustic and vibration signals are both dynamic responses of mechanical components. Vibration measurement directly reflects the structural response, whereas acoustic measurement captures the radiated sound caused by the same mechanical excitation. Therefore, acoustic signals can serve as a non-contact complementary indicator for bearing-state monitoring, especially when contact sensor installation is inconvenient.

**Figure 1:**
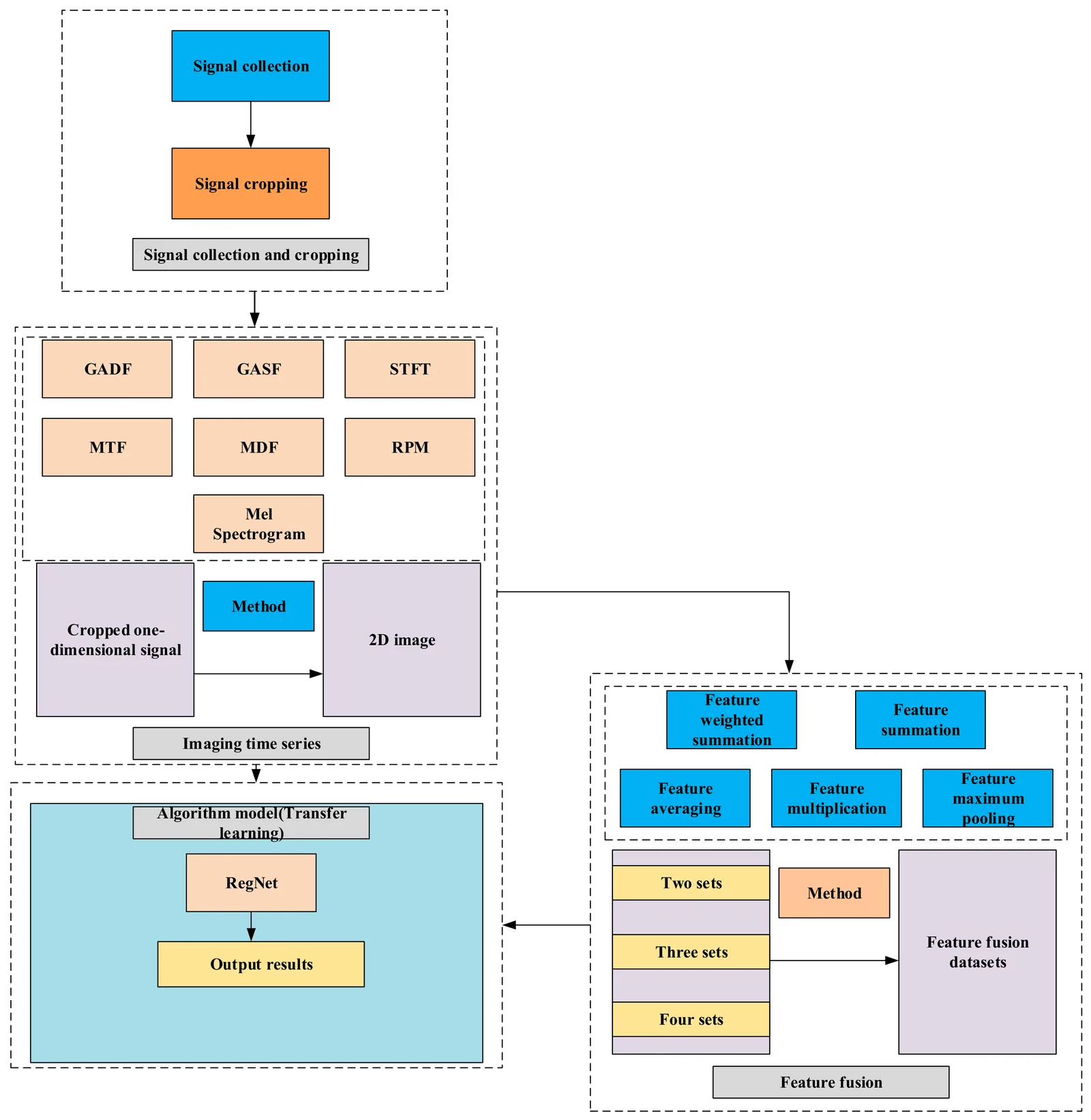
Process of the fault diagnosis of motor bearing transmission system.

This paper focuses on the motor bearing transmission system and introduces a fault diagnosis method that integrates acoustic features. The approach involves segmenting the complete signal and applying seven distinct methods, including Gramian Angular Difference Field (GADF) and Gramian Angular Summation Field (GASF), to generate time series images from the one-dimensional signal. These images are then fed into a transfer learning network utilizing the RegNet architecture. Datasets that ranked highest in accuracy (top two, three, and four) are chosen for feature fusion, creating new datasets. Subsequently, these fused datasets are utilized for training to improve bearing fault diagnosis accuracy. The representation of this paper is depicted in Figure 1.

The rest of this paper is organized as follows: Section 2 presents the methodology and principles. Section 3 gives the details of the experimental setup and datasets. Section 4 describes the proposed algorithm and how it was trained and describes the information on the feature fusion datasets and the analysis of the results obtained using them as input. Finally, Section 5 draws the conclusion.

## 2. Materials and Methods

### 2.1. RegNet Network

The RegNet network was a new network design paradigm proposed by the Facebook AI research team in 2020. Rather than concentrating on designing a single network, they conceptualized a network design space tailored for parameterized network groups aimed at exploring various possibilities for network architecture design and finding the optimal network design rules [19].

The design of RegNet is based on the following two observations: the depth (number of layers), width (size of each layer), and resolution (size of input data) of the network have a great impact on network performance, and there is a strong correlation among them; the network structure that achieves excellent performance usually has a certain “rule” in which the ratio of parameters such as depth, width, and resolution meets certain rules [19].

The design concept and implementation of RegNet provide a new idea for the design of convolutional neural network architecture, which is more flexible and efficient than traditional manual design and automated search. The network model framework has shown good performance in visual tasks such as ImageNet classification tasks.

### 2.2. Convolutional Neural Networks

A convolutional neural network, or CNN for short, is a feedforward neural network designed to process grid-structured data, like two-dimensional image data, by emulating the structure and functionality of biological visual systems. It has demonstrated significant success in various domains such as computer vision, image recognition, and video analysis [20,21]. CNNs have the capability to extract both traditional topological structural features of samples and high-dimensional abstract features that are challenging to represent using traditional mathematical models [16].

The main components of a convolutional neural network include the following:

The input layer, as the first layer of the convolutional neural network, plays a fundamental role by receiving data for learning.

The convolutional layer, the key component of convolutional neural networks, uses a sliding window, known as a convolution kernel, to extract features from local areas in the input data by performing mathematical operations. Through convolutions, a convolution kernel, comprising learnable weight matrices, is employed to capture local patterns and spatial features of the input image.

The output of the convolution operation is expressed as:

(1) $$O(i,j)=(I*K)(i,j)=\sum_{m}\sum_{n}I(m,n)K(i-m,j-n)$$

Among them, * represents the convolution operation, *K* is the kernel function of the convolution operation, *m* and *n* are the step sizes, which means that the convolution kernel is shifted to the right and downward by *m* and *n* units respectively.

Activation functions, such as the Sigmoid function, Tanh function, ReLU function, among others, serve to introduce nonlinear elements into neural networks. By incorporating these activation functions, the network can effectively learn complex patterns and bolster its capacity for nonlinear expression, thereby enabling it to tackle intricate problems that require nonlinear solutions. One of the most frequently utilized activation functions is the ReLU activation function, which is typically expressed as follows:

*f*(*x*) = *max*(0,*x*). (2)

Among them, *x* is the input and *f*(*x*) is the output.

The pooling layer in a neural network serves several key functions. This layer achieves dimensionality reduction and extracts dominant features by downsampling local areas, which helps prevent the model from overfitting. Common pooling operations, such as maximum pooling and average pooling, are utilized for this purpose. By reducing the number of parameters, the pooling layer enhances computational efficiency and exhibits a degree of displacement invariance.

The fully connected layer flattens the feature vectors from the convolutional and pooling layers, then connects them to the fully connected network layer for combining and classifying high-level features.

This convolutional neural network architecture has demonstrated significant capabilities in capturing advanced and complex features layer by layer while maintaining spatial correlation, which in turn enhances its effectiveness in image recognition and classification tasks. Because it can effectively reduce the number of parameters and better extract spatial information, it has shown good performance in processing tasks such as images and speech [22,23,24].

### 2.3. Feature Fusion

In the field of machine learning and pattern recognition, feature fusion plays a crucial role. It involves the integration of features originating from various sources or representations to develop a more sophisticated and discriminatory feature representation. The primary objective of feature fusion is to leverage the diverse information offered by heterogeneous feature sources to address the shortcomings of utilizing a single feature representation effectively. As different features are associated with distinct patterns, fusing them together enables a more thorough capture of data distribution characteristics. Consequently, this process enhances the model’s generalization capacity and discriminative performance [25,26,27,28].

Feature fusion can usually be divided into several levels: data-level fusion, feature-level fusion, score-level fusion, and hybrid fusion [29,30]. Common feature fusion methods include vector concatenation, tensor concatenation, weighted summation, multiple ERP fusion, and maximum pooling [29,31,32].

Reasonable feature fusion strategies can fully explore the complementary patterns in heterogeneous data sources and improve the final task performance [25,26,27,28].

## 3. Experimental Setup and Dataset Construction

### 3.1. Bearing Defect Fault Setting

**Figure 2:**
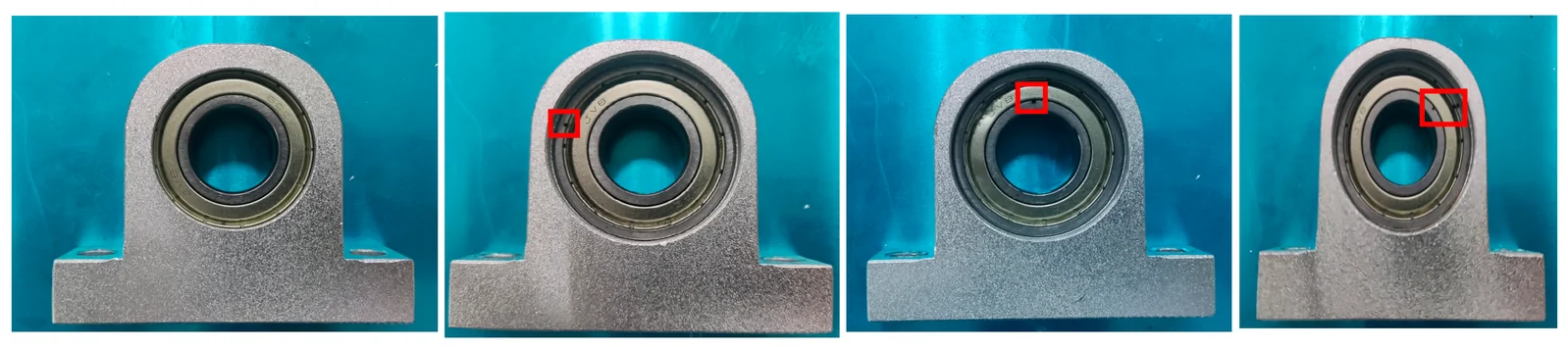
Bearings (from left to right are non-defective intact bearing, outer ring defect bearing, inner ring defect bearing, and bearing with mixed internal and external defects). The red boxes indicate the machined defect positions.

To address the bearing fault diagnosis problem of the motor bearing transmission system using acoustic signals, the initial step involves selecting a suitable bearing and inducing defects. For this study, the P6004 closed deep-groove ball bearing was selected, with an inner diameter of 20 mm. Hole-type defects were machined on the inner ring and outer ring of the bearing, respectively. The defect diameter was 0.5 mm, and the defect depth was 12 mm. Four bearing states were considered in this study: non-defective intact bearing, outer ring defect bearing, inner ring defect bearing, and bearing with mixed inner and outer ring defects. The bearing type and defect size were kept fixed to ensure the consistency of the acoustic acquisition process and to focus on the feasibility of the acoustic feature fusion method. The influence of different bearing types and defect severities will be further investigated in future work. These situations are visually represented from left to right in Figure 2. Because the defects were artificially machined, the experimental results mainly demonstrate the feasibility of the proposed method under the present experimental setup, while further validation using naturally evolved bearing faults remains necessary.

### 3.2. Laboratory Bench Setup

**Figure 3:**
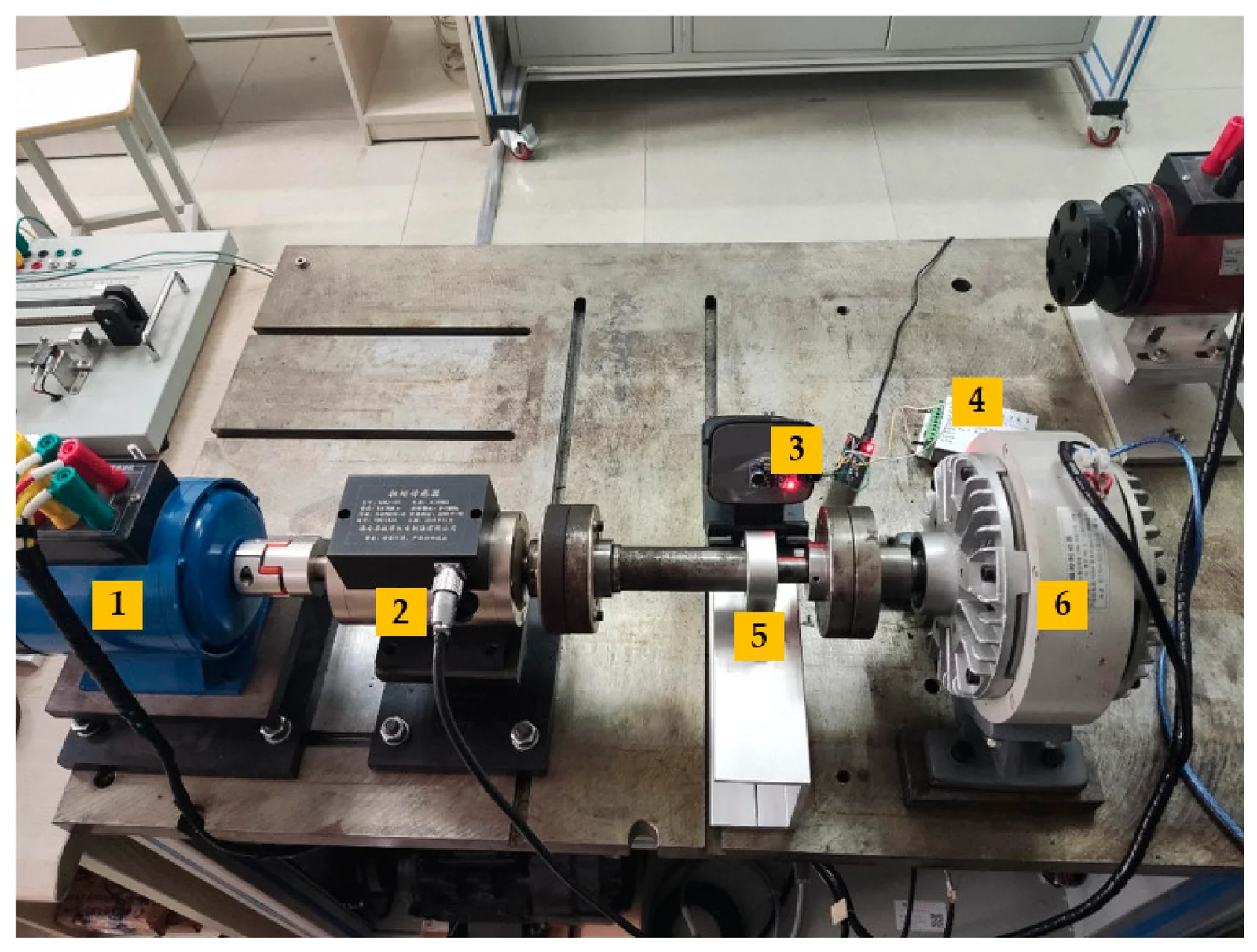
The entire experimental platform.

The overall layout of the experimental platform for this task is shown in Figure 3. Among them, 1 is a three-phase asynchronous motor, 2 is a torque sensor, 3 is an acoustic sensor, 4 represents the data acquisition card, 5 denotes the bearing and bearing seat, and 6 corresponds to the magnetic powder brake. More specifically, the experimental platform consisted of an HKDJ-26 three-phase asynchronous motor (custom experimental motor; manufacturer information unavailable), an HCNJ-101 torque sensor (Hai’an Hangyu Electromechanical Manufacturing Co., Ltd., Hai’an, China), an LM386 acoustic sensor (manufacturer information unavailable), a DAQ122 data acquisition card (Fuzhou Lingzhi Electronic Co., Ltd., Fuzhou, China), a bearing seat, and a CZ-2 magnetic powder brake (Jiangsu Hangyu Electromechanical Manufacturing Co., Ltd., Hai’an, China). The main parameters of the acoustic sensor are added to improve the reproducibility of the experimental setup. During data acquisition, the acoustic sensor was kept fixed relative to the bearing seat so that all bearing states were collected under the same sensor-position condition. The detailed parameters are shown in Table 1.

### 3.3. Sound Signal Collection Settings

In the experiment, the motor speed was set to remain at 500 rpm with no load applied. The sampling rate for sound acquisition was set at 48 kHz.

### 3.4. Signal Processing and Parameter Setting

**Figure 4:**
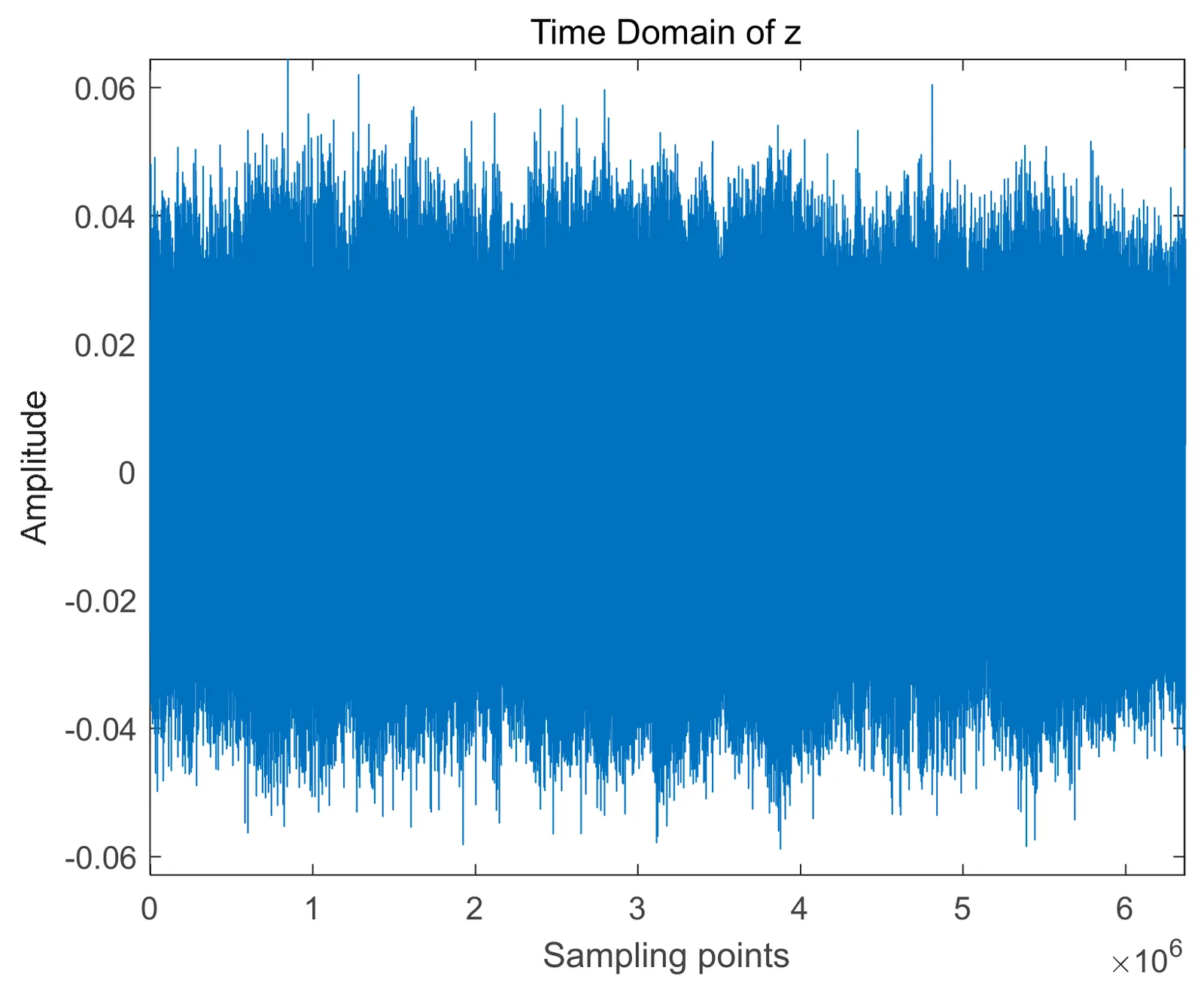
Sound signal of non-defective intact bearing.

**Figure 5:**
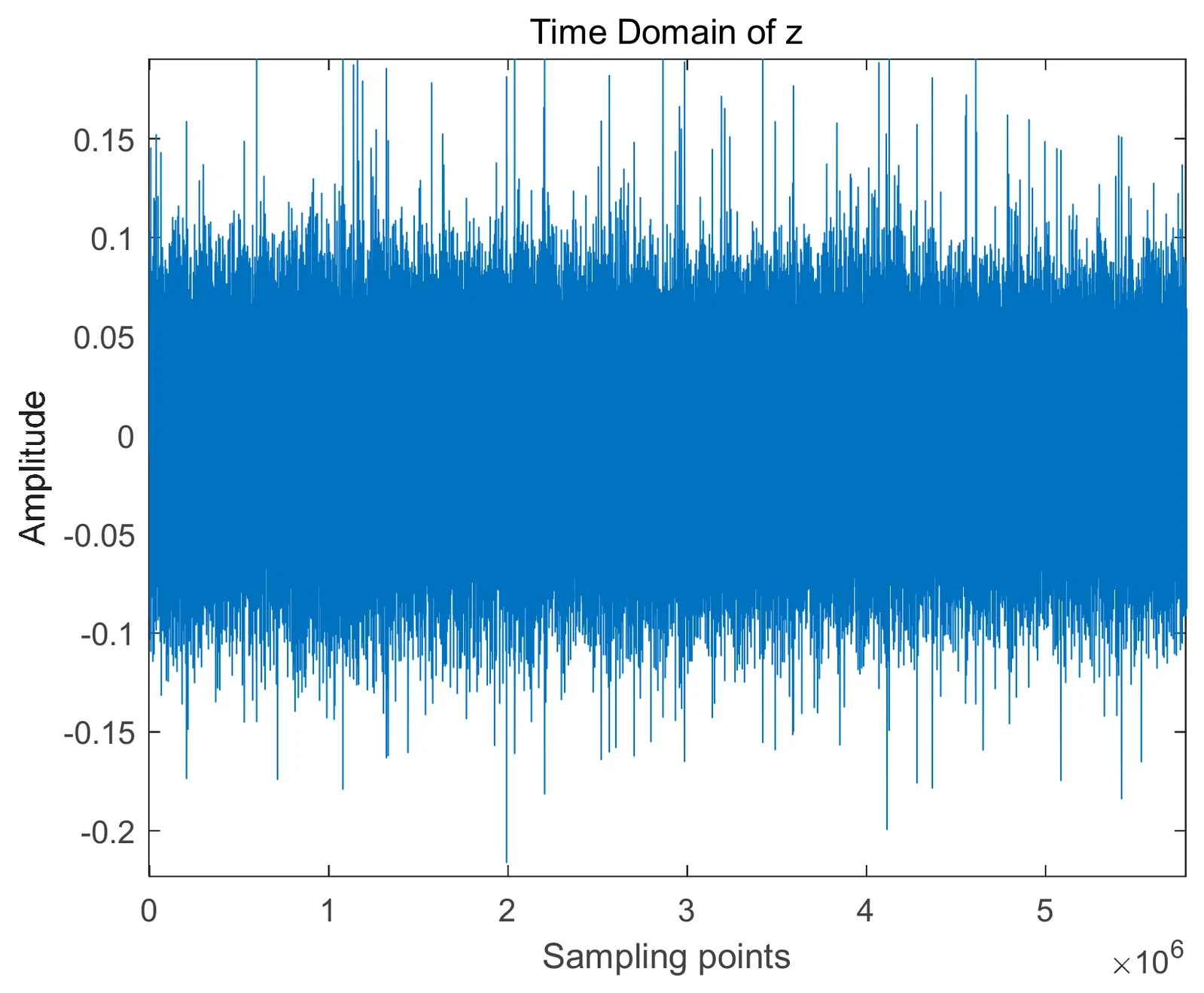
Sound signal of outer ring defect bearing.

**Figure 6:**
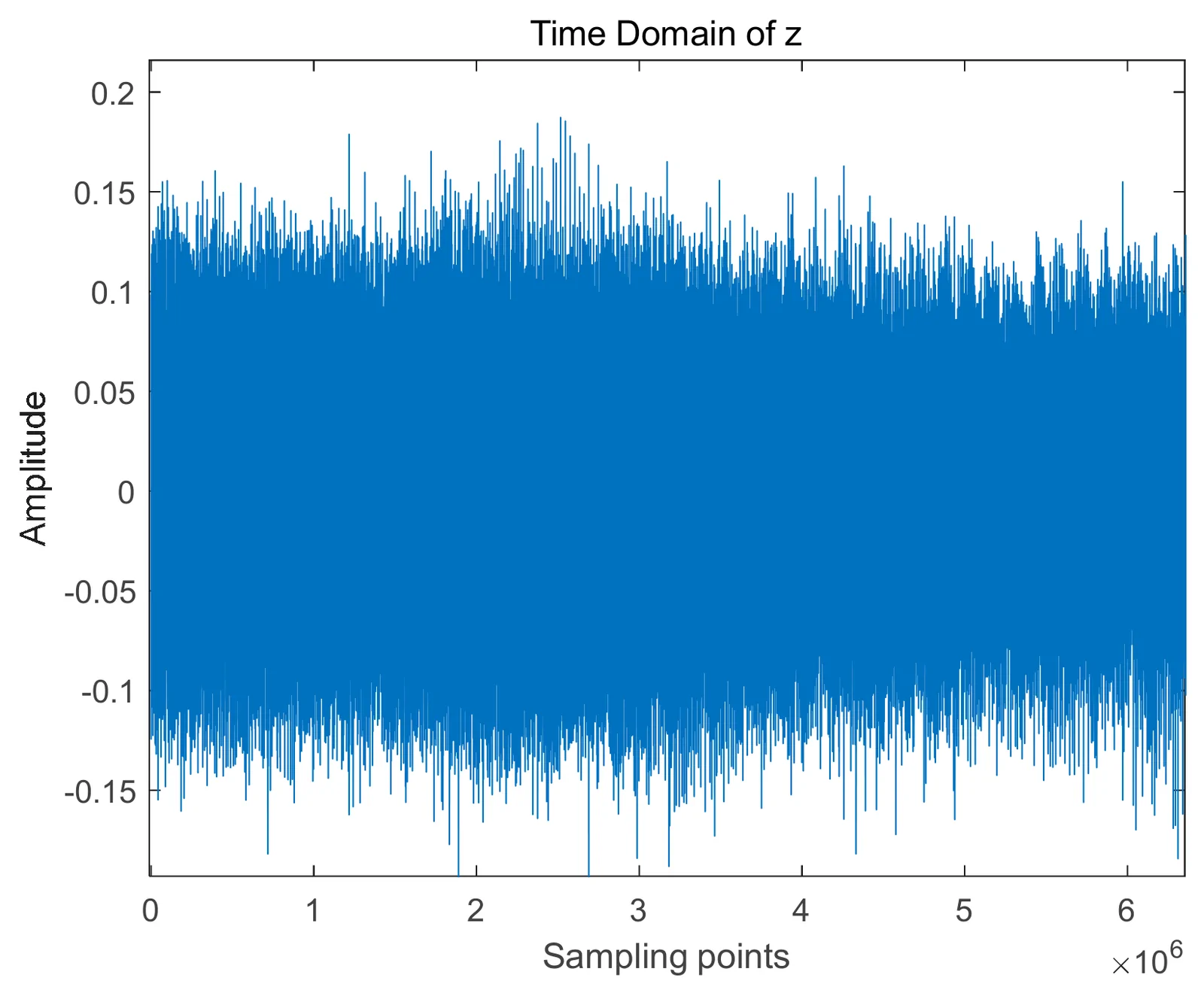
Sound signal of inner ring defect bearing.

**Figure 7:**
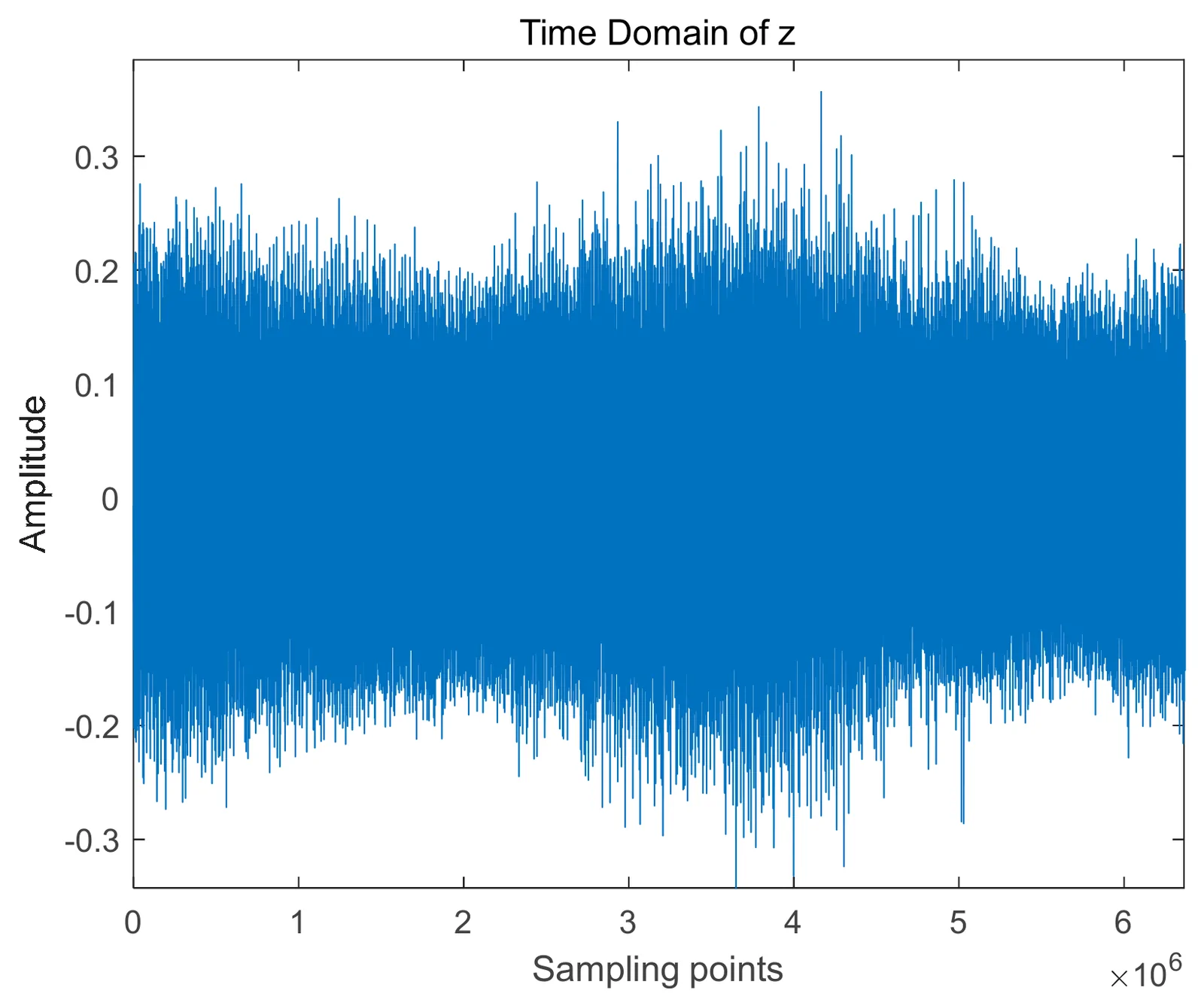
Sound signal of bearing with mixed internal and external defects.

In this experiment, sound signals of the transmission system were collected under four different conditions: non-defective intact bearing, outer ring defect bearing, inner ring defect bearing, and bearing with mixed internal and external defects. Figure 4, Figure 5, Figure 6 and Figure 7 display the time-domain representation of the four signal groups, sequenced from top to bottom as follows: the signal of the non-defective intact bearing, the signal of the outer ring defect bearing, the signal of the inner ring defect bearing, and the signal of the bearing with mixed internal and external defects.

In order to facilitate further processing, the periodic nature of the collected sound signal necessitates the segmentation of the entire signal into multiple segments. The sampling frequency was 48 kHz, and the motor speed was 500 rpm, corresponding to approximately 8.33 rotations per second. Therefore, one complete rotation required about 0.120 s, i.e., approximately 5760 sampling points. The segment length was set to 6100 sampling points to ensure that each segmented acoustic signal contains one complete bearing rotation. This setting ensures that each segment contains at least one complete rotation cycle and provides a small margin to avoid cutting the fault-related acoustic response exactly at the segment boundary. The overlap length was set to 0 during segmentation. In other words, non-overlapping segmentation was adopted to reduce the similarity between adjacent samples as much as possible. After segmentation, the same segment indices were used for all time-series imaging methods to ensure a consistent comparison among different feature datasets.

After the signal is trimmed, the datasets can be constructed.

### 3.5. Dataset Construction

After the above processing, each set of complete one-dimensional signals is segmented into a series of segmented signals. A one-dimensional time signal can be called a one-dimensional time series. A time series refers to a record of the process of event changes and developments in chronological order, which preserves the temporal structure of the observed data [33].

In recent years, deep learning has experienced rapid development and is increasingly being employed in various fields. Scholars, inspired by the success of Convolutional Neural Networks (CNNs) in image classification tasks, have suggested a novel approach: transforming one-dimensional time series data into two-dimensional images. By leveraging this technique, time series classification tasks can be effectively reframed as image classification challenges. The resulting two-dimensional images are then utilized as input for training and learning within the CNN model [34,35,36]. We also continue our research based on this idea.

There are many methods for imaging time series. From the perspective of time-frequency analysis, there are Short-Time Fourier Transform, Wavelet Transform, etc.; from the perspective of image coding, there are Gramian Angular Field, Markov Transition Field, Motif Difference Field, Relative Position Matrix, etc. [34,35,36]. Since we collect sound signals, we can also convert the one-dimensional time series into a two-dimensional Mel Spectrogram [16]. In this paper, we use seven methods, including Gramian Angular Field, Short-Time Fourier Transform, and Mel Spectrogram, to realize imaging time series. The specific methods and their explanations are shown in Table 2.

**Figure 8:**
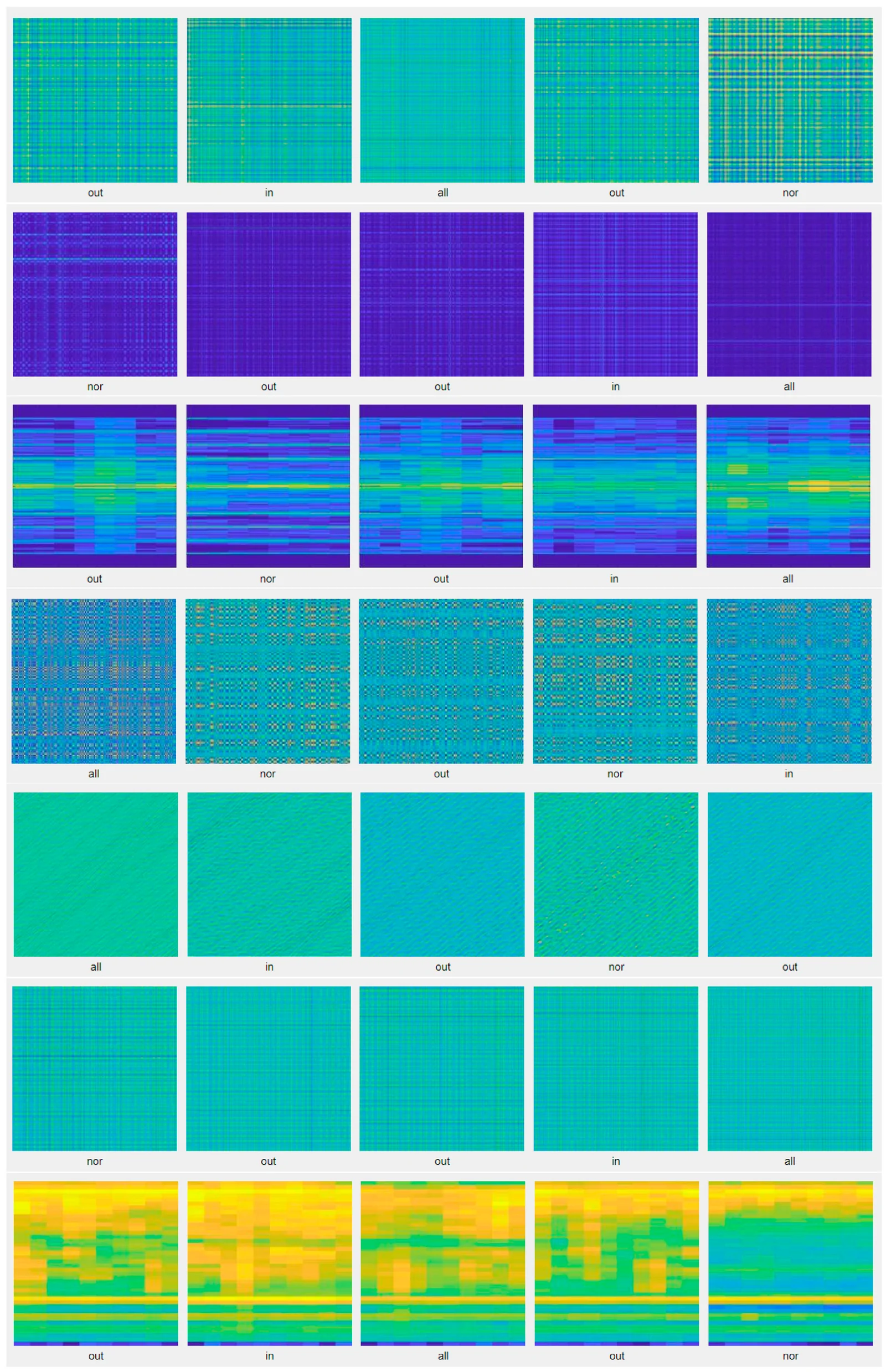
Partial images display (from top to bottom are GADF; GASF; STFT; MTF; MDF; RPM; Mel spectrogram). The colors in the feature maps are used to visualize the relative values of the transformed acoustic features and do not represent physical color information.

This paper uses the above seven methods to realize time-series imaging of the cropped signal to obtain the corresponding two-dimensional feature maps. To improve reproducibility, the key processing procedure was clarified as follows: each acoustic sample contained 6100 sampling points; each feature image was generated from the same segment index; the generated images were resized to 224 × 224 and converted into RGB images. A total of 1059 images were generated for each bearing state. This number was determined by the available length of each complete acoustic recording, the 6100-point non-overlapping segmentation rule, and the removal of the incomplete tail segment. No additional data augmentation was used to increase the number of samples. The specific information of the datasets is shown in Table 3. Some images are shown in Figure 8. Among them, nor represents the non-defective intact bearing, out represents the outer ring defect bearing, in represents the inner ring defect bearing, and all represents the bearing with mixed internal and external defects.

The seven time-series imaging methods were selected to include two different representation approaches. STFT and Mel spectrogram describe the acoustic signal from the perspective of time-frequency distribution, whereas GADF, GASF, MTF, MDF, and RPM encode the time series into image representations from the perspective of temporal dependence or transition relationships. Therefore, GAF representations provide a different image-encoding perspective from STFT rather than simply repeating the same frequency-domain information.

## 4. Fault Diagnosis of Motor Bearing Transmission System Based on Transfer Learning and Feature Fusion of RegNet Model

### 4.1. Construction of Algorithm Model

According to the detailed description of the datasets, it is not difficult to see that compared with the common large-scale image processing datasets (such as ImageNet), this task is a small-sample problem. To address the small sample problem, we use transfer learning-based fine-tuning to perform fault diagnosis tasks on motor bearing transmission systems.

Regarding the selection of the model, this experiment uses the RegNet network model as the pre-training model for this task.

To justify the selection of RegNet as the network, several representative transfer learning models were compared in the preliminary stage of this study using the GADF dataset under the same training settings. The final classification layer of each pre-trained model was modified to output four bearing states. The compared models included EfficientNet, EfficientNetv2, MobileNet, RegNet, and SqueezeNet. The training/test ratio was 7:3, the batch size was 32, the epoch number was 250, the initial learning rate was 0.001, the Adam optimizer was used, and cross-entropy was adopted as the loss function. Mixed precision training was used to improve training efficiency. As shown in Table 4, RegNet achieved the highest accuracy among the compared networks. Therefore, RegNet was selected as the network for the subsequent single-feature and feature-fusion experiments.

The specific steps are as follows:

First, load the pre-trained model;

Next, adjust the final layer of the model to modify the classification to 4 in order to suit the current task;

Finally, use the above seven methods to implement the imaging time series to obtain the corresponding two-dimensional spectrum-generated datasets for training.

### 4.2. Equipment Environment Configuration

The computer hardware environment parameters and computer software environment parameters used in this paper are shown in Table 5 and Table 6.

### 4.3. Single Feature Results and Analysis

In the parameter setting, the ratio of training set to test set is about 7:3 (748:311). Batch size is 32, epoch is 250, learning rate is 0.001, Adabelief optimizer and MultiStepLR dynamic learning rate method are used, parameters are 50, 100, 150, gamma is 0.1, cross-entropy is used as the loss function, and RegNet uses the regnety_040 variant. In order to speed up training, mixed precision training is used. The training results of the datasets obtained by seven methods to implement imaging time series are shown in Table 7. The reported training time refers to the network training stage and does not include the offline time-series image generation stage; therefore, the training times of different imaging datasets mainly reflect the cost of training the same RegNet model on images with the same input size.

The choice of the optimizer and learning rate strategy was also based on preliminary comparisons. Under RegNet, Adabelief achieved better diagnostic accuracy than Adam, AdamW, Lookahead, and Radam in the preliminary evaluation. Different learning rate decay strategies were further compared under the Adabelief optimizer. MultiStepLR was finally adopted because it provides staged learning rate adjustment and stable training behavior in the subsequent feature-comparison experiments.

**Figure 9:**
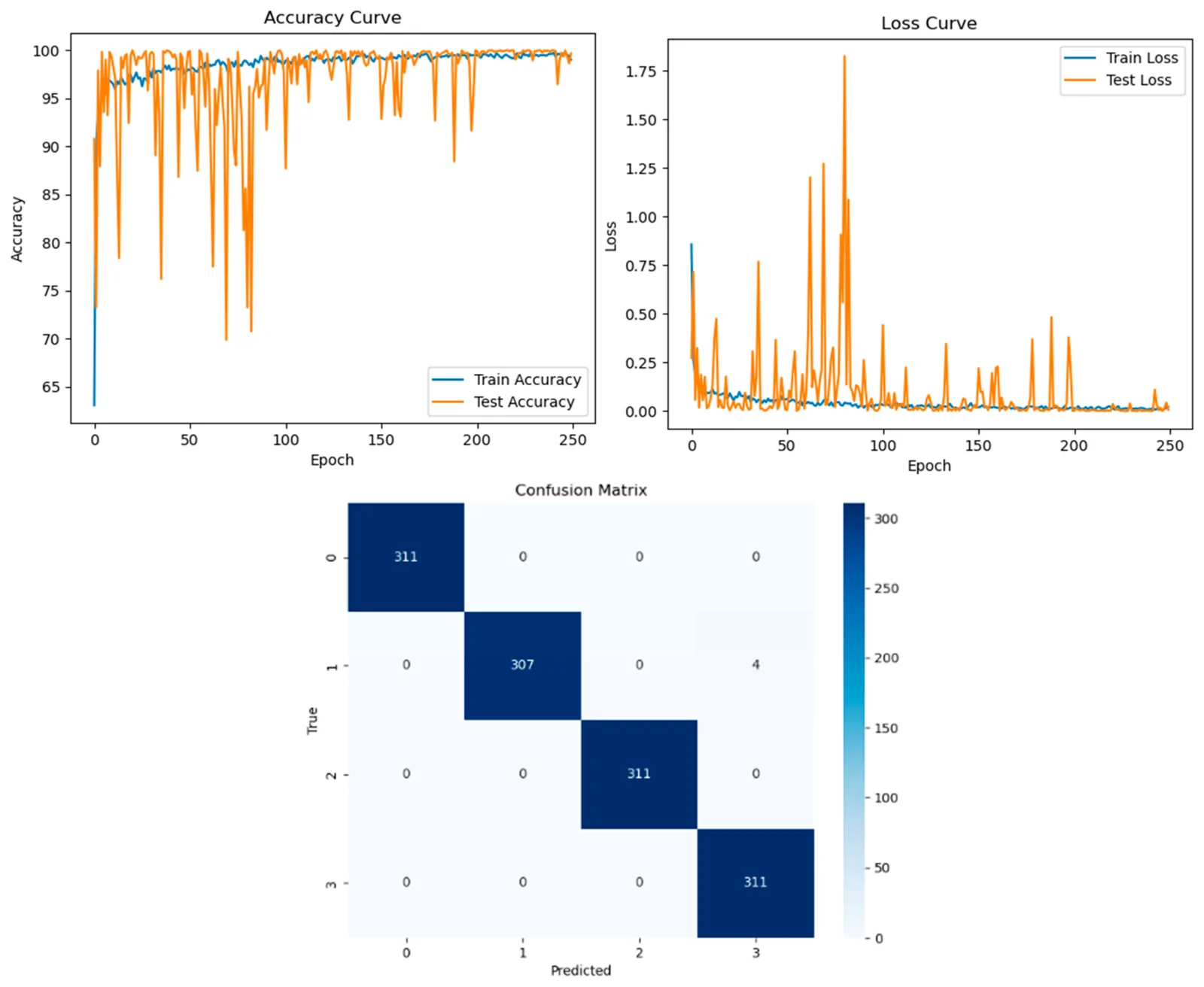
Accuracy curve, loss function curve, confusion matrix of STFT.

**Figure 10:**
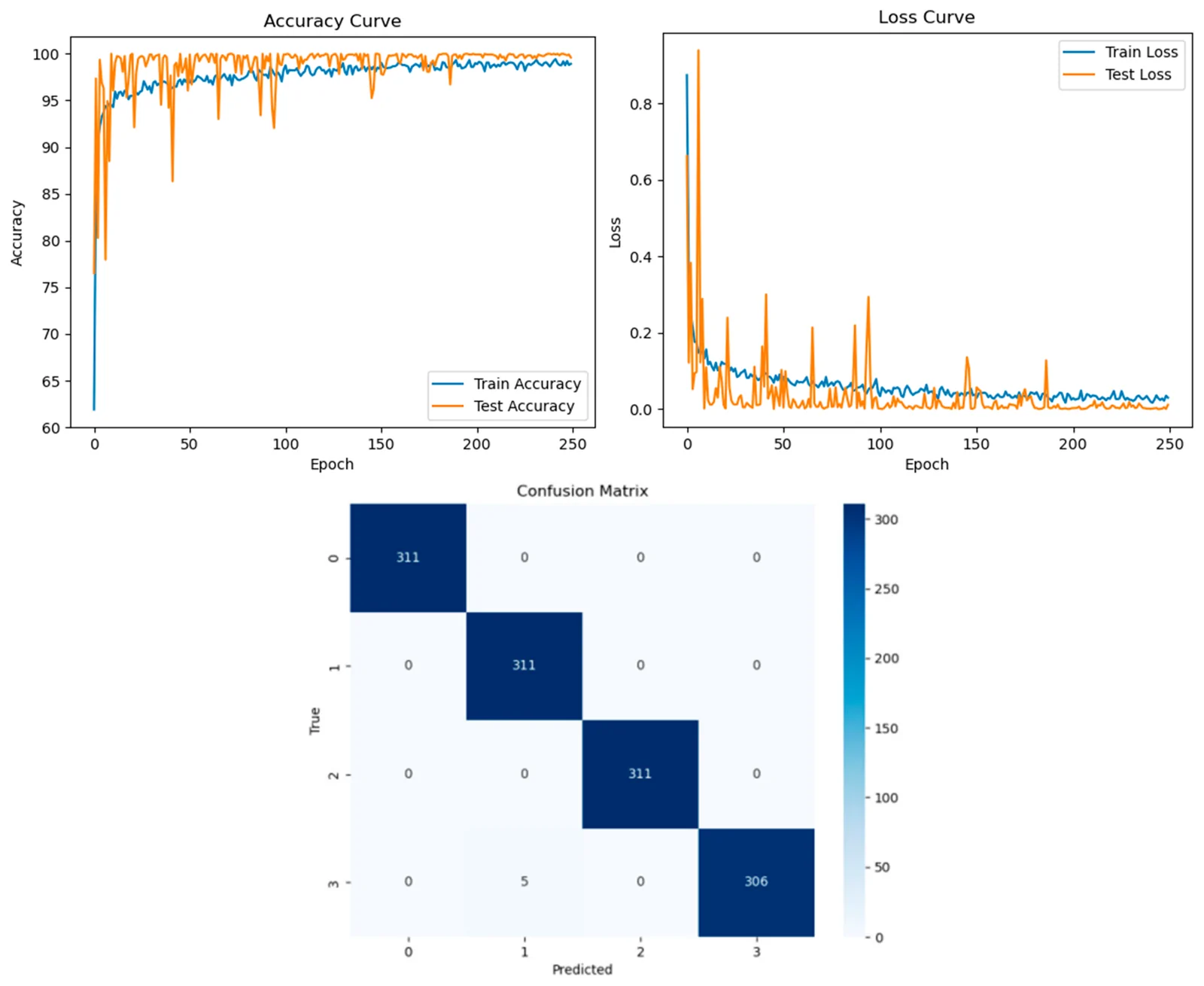
Accuracy curve, loss function curve, confusion matrix of Mel Spectrogram.

Through the above comparison, considering the accuracy and training time of the results, the training results of STFT and Mel Spectrogram are initially selected. Next, further analysis is performed through the accuracy and loss function curves and the confusion matrix. The comparison results are shown in Figure 9 and Figure 10. Among them, 0 represents the bearing with mixed internal and external defects (all); 1 represents the inner ring defect bearing (in); 2 represents the non-defective intact bearing (nor); and 3 represents the outer ring defect bearing (out).

By comparing the results, we can see that although the STFT dataset has the highest accuracy, the accuracy curve and loss function curve of the Mel spectrogram dataset are more stable. Both datasets achieved an accuracy of over 99.60% under the present experimental setup, indicating that both STFT and Mel spectrogram features contain discriminative information for the four bearing states.

The next question is whether the existing research can improve accuracy further. For this purpose, the feature fusion method is used to combine multiple pieces of information from different feature sets for retraining.

### 4.4. Feature Fusion and Fusion Datasets Construction

The five fusion strategies were implemented at the image level and are defined in Table 8. Let *X_k_* denote the k-th feature image after resizing, where *k* = 1, 2, …, *K*, and let *X_f_* denote the fused image. The fusion operations were performed pixel by pixel. In the weighted-summation strategy, the weight of each feature image was determined according to its single-feature diagnostic accuracy before fusion.

**Figure 11:**
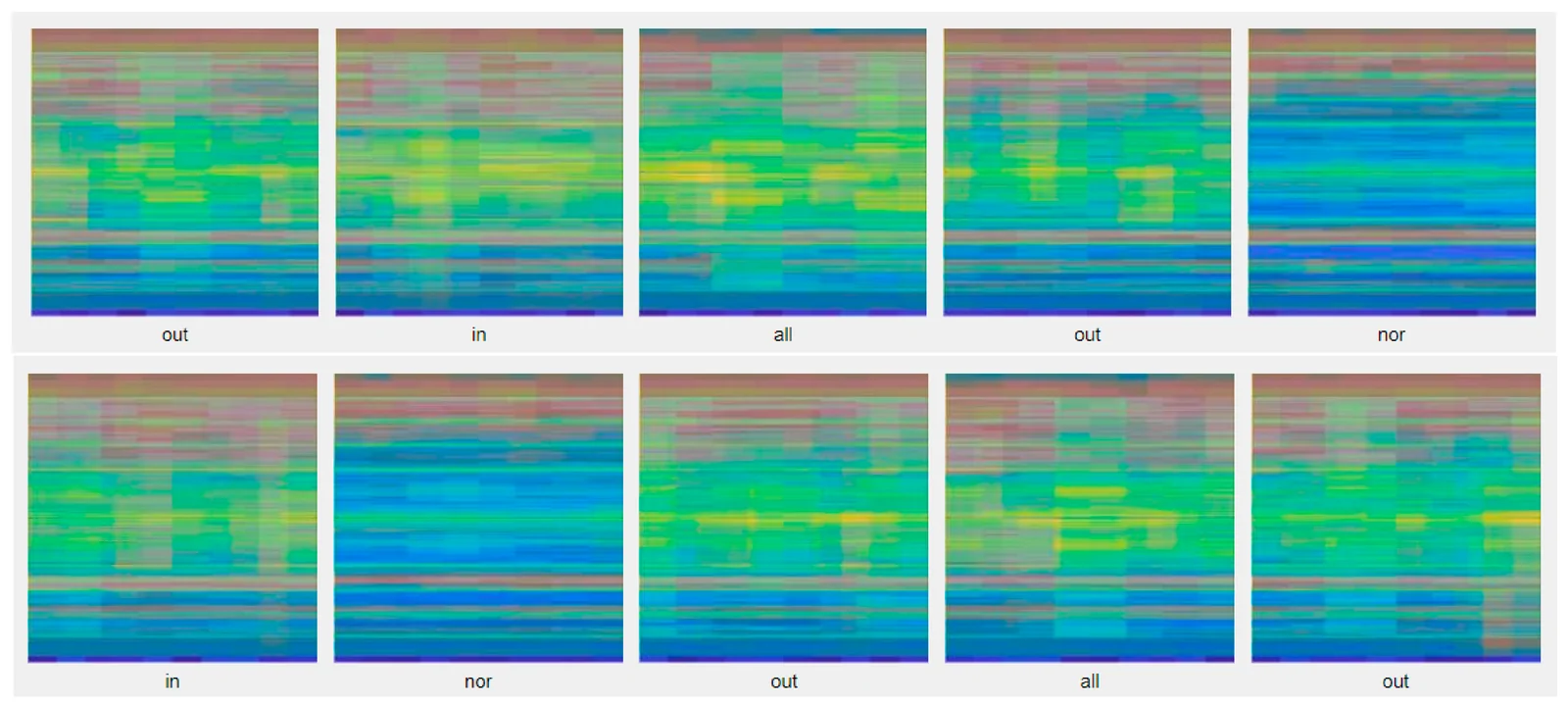
Partial image displays of feature fusion datasets (two sets for feature-weighted summation and feature averaging). The colors in the feature maps are used to visualize the relative values of the fused acoustic features and do not represent physical color information.

**Figure 12:**
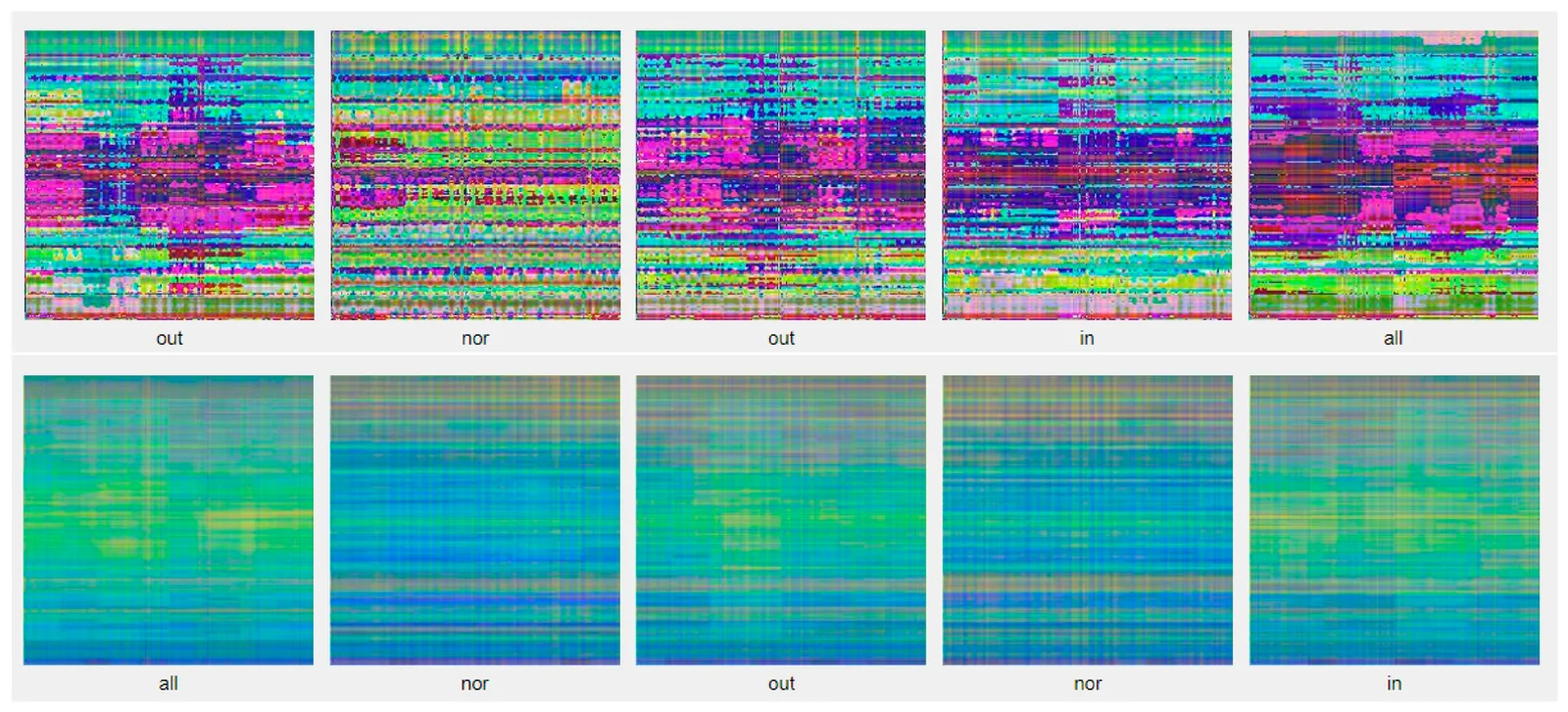
Partial image displays of feature fusion datasets (three sets for feature summation and feature averaging). The colors in the feature maps are used to visualize the relative values of the fused acoustic features and do not represent physical color information.

**Figure 13:**
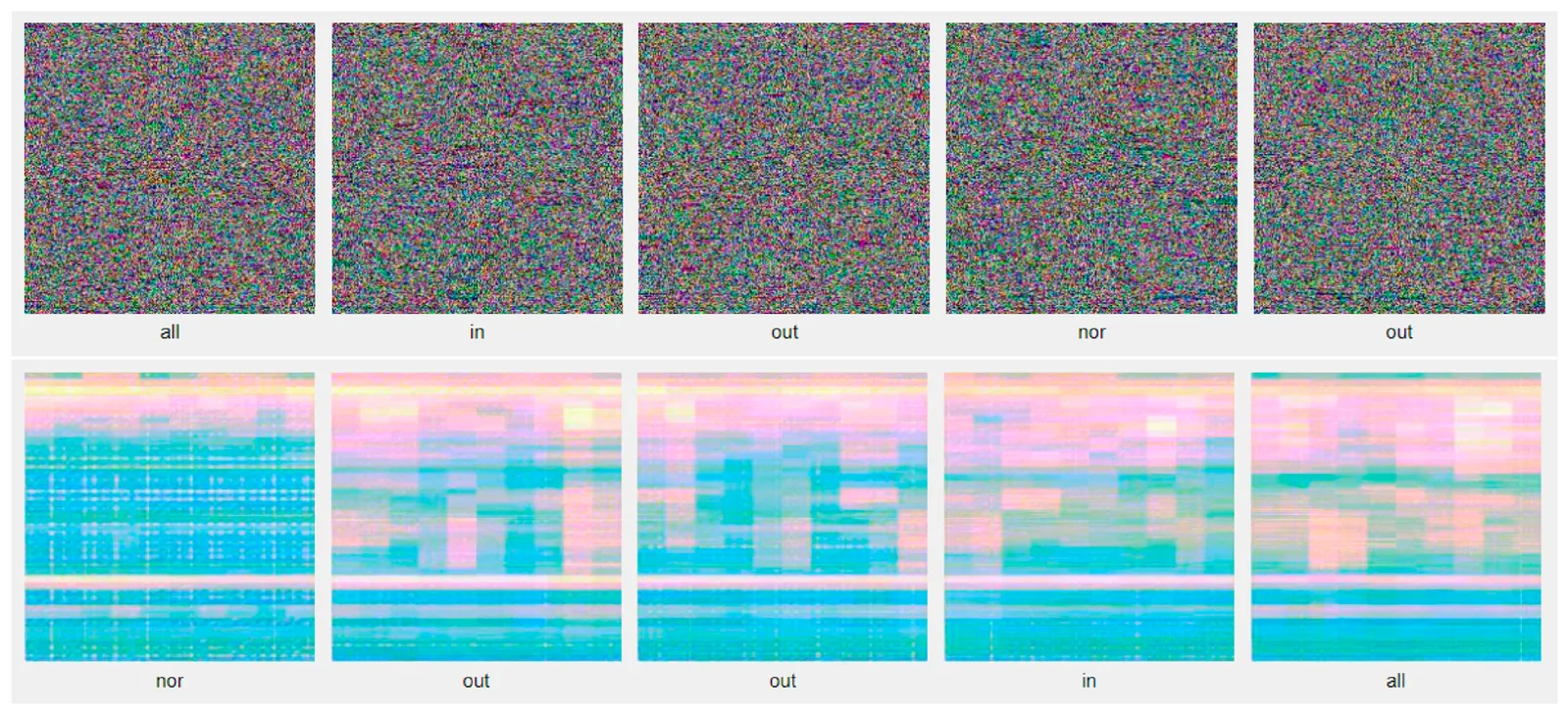
Partial image displays of feature fusion datasets (four sets for feature multiplication and feature maximum pooling). The colors in the feature maps are used to visualize the relative values of the fused acoustic features and do not represent physical color information.

We took the seven single feature datasets mentioned above and sorted them by accuracy. The top two, three, and four datasets in terms of accuracy were selected for further analysis. To construct new fusion feature datasets, we employed five different methods: feature weighted summation, feature summation, feature averaging, feature multiplication, and feature maximum pooling. Table 9, Table 10 and Table 11 provide detailed information on the specific characteristics of these datasets. Due to the large number of datasets, here we show the datasets implemented by two feature fusion methods for three sets of feature datasets with different numbers of single features, as shown in Figure 11, Figure 12 and Figure 13. Although the number of images in each bearing state and the image size are the same for all fusion datasets, Table 9, Table 10 and Table 11 were retained separately to clearly present the feature combinations and fusion strategies used for the two-feature, three-feature, and four-feature fusion datasets.

Keeping the same parameter settings as above, 15 sets of feature fusion datasets are trained. The results are shown in Table 12, Table 13 and Table 14.

### 4.5. Feature Fusion Results and Analysis

**Figure 14:**
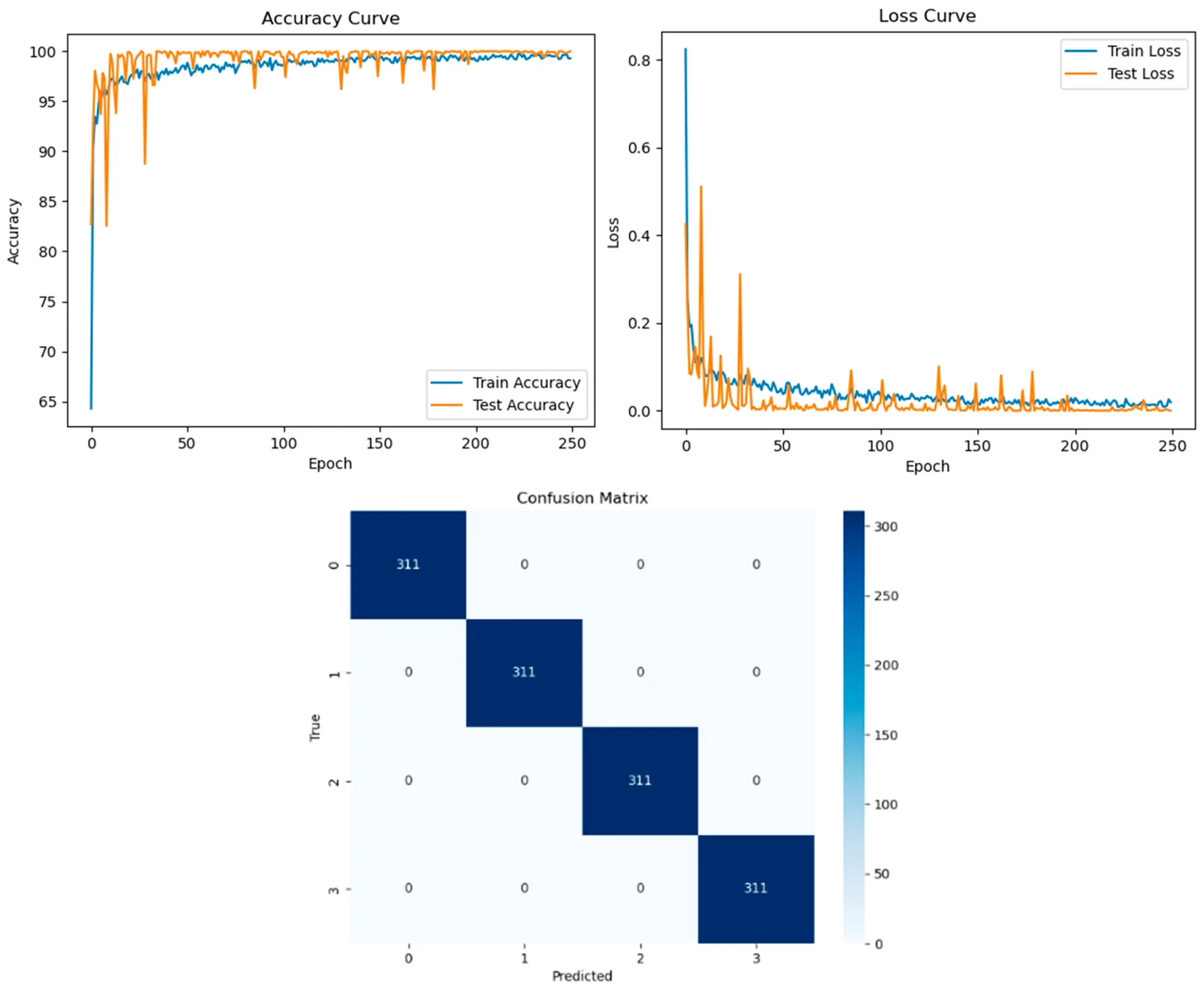
Accuracy curve, loss function curve, confusion matrix of summing up two sets of features.

**Figure 15:**
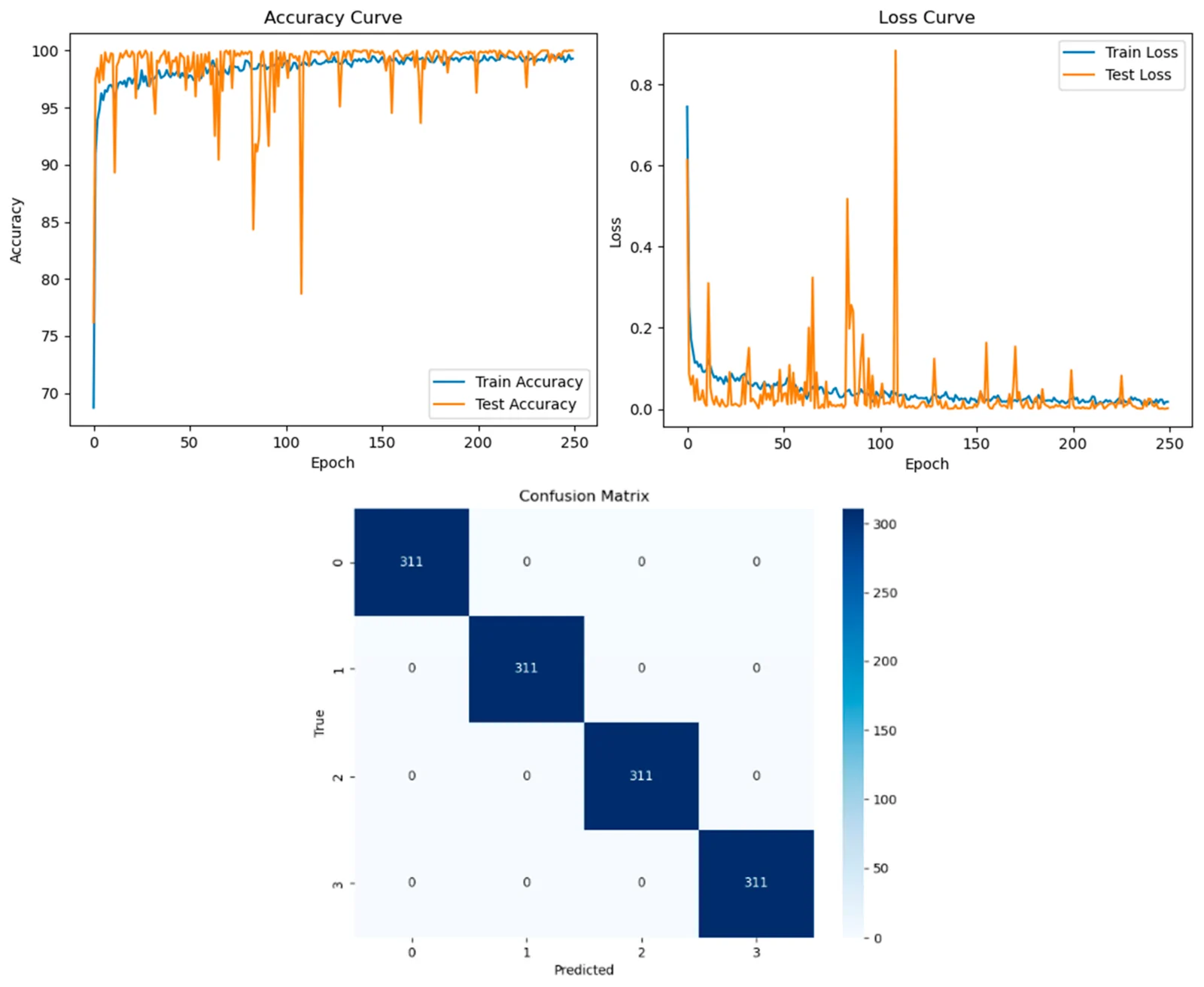
Accuracy curve, loss function curve, and confusion matrix of maximizing feature pooling using four sets of features.

After observing and analyzing the results of the 15 feature fusion datasets mentioned above, it is found that all training results do not significantly differ in terms of processing time, with time fluctuations falling within an acceptable range. However, there are variations in accuracy, as some feature fusion datasets exhibit a decrease in accuracy while others demonstrate higher accuracy compared to using a single feature. Notably, the dataset achieves 100.00% accuracy when performing a feature summation operation on two sets of STFT and Mel Spectrogram datasets. Similarly, the dataset attains 100.00% accuracy when engaging in a feature maximum pooling operation on four sets of datasets, with the latter exhibiting a slightly shorter training time than the former. Subsequently, a more in-depth analysis is conducted using the accuracy and loss function curves alongside the confusion matrix. The comparison outcomes are illustrated in Figure 14 and Figure 15. Among them, 0 represents the bearing with mixed internal and external defects (all); 1 represents the inner ring defect bearing (in); 2 represents the non-defective intact bearing (nor); and 3 represents the outer ring defect bearing (out).

The accuracy and loss function curves and confusion matrices of the two fusion strategies are comprehensively compared. It can be observed that both strategies achieved an accuracy of 100.00% under the present experimental setup and dataset partition. However, the training results of the fusion feature dataset using two sets of features for feature summation are more stable than those of the fusion dataset using four sets for feature maximum pooling, and the training time of the two strategies is similar. In summary, the fusion feature dataset generated by summing two feature maps, namely STFT and Mel spectrogram, was selected as the final input dataset in this study. The obtained accuracy was 100.00%, and the training time was 10,186.37 s. STFT and Mel spectrogram are two different time-frequency representation methods. STFT represents the acoustic signal based on a linear frequency distribution, whereas the Mel spectrogram represents the acoustic signal based on the Mel frequency scale. In this study, the STFT + Mel spectrogram summation dataset achieved the best diagnostic performance among the tested fusion datasets. Therefore, this dataset was selected as the final input in this study.

### 4.6. Discussion and Future Work

Although the proposed acoustic feature fusion method achieved high diagnostic accuracy in the current experiment, several aspects require further investigation in future work.

First, the experiments in this study were conducted at a fixed rotating speed of 500 rpm and under a no-load condition. Acoustic signals are sensitive to operating conditions, such as rotating speed, load, sensor position, background noise, and defect severity. Therefore, the current results mainly demonstrate the feasibility of the proposed method under the present experimental setup. Future work will collect data under different speeds, load levels, sensor positions, and background noise levels to further evaluate the robustness of the proposed method.

Second, although four bearing states were considered, including the normal state, outer-ring defect, inner-ring defect, and mixed inner- and outer-ring defects, only one bearing model and one artificially machined defect geometry were used in this study. The defect diameter and depth were fixed at 0.5 mm and 12 mm, respectively. Future work will further investigate other bearing models, naturally evolved faults, and different defect severities.

Third, the acoustic sensor position was kept fixed in the current experiment. The influence of sensor distance, sensor orientation, calibration uncertainty, and environmental noise was not systematically evaluated. Therefore, future work will analyze the influence of different sensor installation conditions and improve the repeatability of acoustic signal acquisition.

Fourth, RegNet was selected as the backbone network based on a preliminary comparison of several image-based transfer-learning networks, including EfficientNet, EfficientNetv2, MobileNet, RegNet, and SqueezeNet. Future work will compare more image-based diagnostic networks under larger datasets and different operating conditions to further evaluate the robustness and applicability of the proposed method.

Fifth, the current dataset was constructed by segmenting continuous acoustic recordings into 6100-point non-overlapping samples. Although non-overlapping segmentation was adopted to reduce the similarity between adjacent samples, the current 7:3 training/test split was still performed at the segmented-sample level. Therefore, segments originating from the same continuous recording or acquisition run may share similar acoustic environments, sensor positions, background noise levels, and operating states. This may lead to potential data leakage. In future work, stricter validation strategies, such as leave-one-recording-out, leave-one-run-out, leave-one-bearing-out, or acquisition-session-level splitting, will be adopted to evaluate the diagnostic generalization ability of the proposed method.

Sixth, the current results were obtained based on a single dataset partition, and repeated experiments with different random seeds, cross-validation, or grouped validation were not conducted in this study. In future work, repeated experiments with different random seeds will be performed, and standard deviations, confidence intervals, precision, recall, F1-score, and confusion matrices will be reported to provide a more rigorous statistical evaluation.

Seventh, because the current dataset is relatively small for deep-learning-based fault diagnosis, overfitting control requires further investigation. In this study, transfer learning, learning-rate scheduling, and mixed precision training were used during model training, but dropout, weight decay, early stopping, and other regularization strategies were not systematically evaluated. Future work will compare different regularization strategies and validation protocols to further assess and reduce the risk of overfitting.

Finally, the 100.00% accuracy obtained by some fusion datasets was observed only under the present experimental setup and current dataset partition. Since no independent-recording split, grouped validation, or repeated statistical evaluation was performed, this result needs to be interpreted cautiously. It does not indicate that feature fusion can universally eliminate inter-class confusion or guarantee the same performance under different acquisition sessions or operating conditions. In particular, although the STFT + Mel spectrogram summation dataset reduced the misclassifications observed in the single-feature result on the current test set, further validation is still needed to determine whether this improvement is stable and fault-related rather than being affected by the current data partition.

From a physical perspective, bearing defects can produce periodic impact-related acoustic components associated with shaft rotation and the contact between rolling elements and defect locations. The current window length was selected to retain at least one complete rotation-related response in each sample. However, BPFO/BPFI-related characteristic components were not explicitly calculated for model interpretation in the present work. This issue will be further investigated in future work.

## 5. Conclusions

This paper focuses on the motor bearing transmission system and utilizes acoustic signals to propose a fault diagnosis method based on acoustic feature fusion. The method involves collecting sound signals from four bearing states: non-defective intact bearing, outer ring defect bearing, inner ring defect bearing, and bearing with mixed internal and external defects. To generate time-series images of one-dimensional signals, seven different methods, such as Gramian Angular Difference Field (GADF) and Gramian Angular Summation Field (GASF), are employed. The resulting two-dimensional images are input into a transfer learning network based on RegNet. According to the training results, datasets that rank in the top two, three, and four in terms of accuracy are selected for feature fusion, leading to new datasets. The new datasets are then utilized for training to perform bearing fault diagnosis. The findings obtained under the present experimental setup indicate that acoustic feature fusion can improve the diagnostic performance compared with using a single feature map. Ultimately, the fusion feature dataset generated by summing two feature maps, namely STFT and Mel spectrogram, was selected as the final input dataset in this study. Under the present experimental setup with a fixed speed of 500 rpm and no external load, this fusion dataset achieved the best diagnostic performance among the tested feature datasets, and the accuracy and loss curves showed relatively stable convergence. The current study mainly verifies the feasibility of acoustic feature fusion for bearing fault diagnosis under the present experimental setup. The generalization ability of the proposed method under different rotating speeds, load levels, sensor positions, background noise levels, bearing models, and defect severities still requires further investigation. In future work, a larger dataset collected under different operating conditions will be established, and more image-based diagnostic networks, such as ResNet, VGG, EfficientNet, MobileNet, and SqueezeNet, will be compared to further evaluate the robustness and applicability of the proposed method.

## Figures and Tables

**Table 1:** Main parameters of the acoustic sensor.

Parameter	Value
Model	LM386
Frequency range	50 Hz–20 kHz
Operating voltage	3.3 V–5.3 V
Length	34.42 mm
Width	18.67 mm

**Table 2:** Seven imaging time series.

Method	Principle Explanation
Gramian Angular Difference Field (GADF)	Gramian Angular Field is an encoding method that combines coordinate transformation and Gramian matrix knowledge to transform time series data into images. It can convert time series data into image data, retaining both the complete information of the signal and its dependence on time [34,37]
Gramian Angular Summation Field (GASF)	Gramian Angular Field is an encoding method that combines coordinate transformation and Gramian matrix knowledge to transform time series data into images. It can convert time series data into image data, retaining both the complete information of the signal and its dependence on time [34,37]
Short-Time Fourier Transform (STFT)	Short-Time Fourier Transform (STFT) is a variation of the Fourier Transform (FT) to confirm the frequency and phase changes in time-varying signals on the time axis [38,39].
Markov Transition Field (MTF)	Markov Transition Field (MTF) is a time series image encoding method based on a Markov transition matrix. This method treats the time series as a Markov process; that is, under the condition that the current state is known, its future evolution does not depend on its past evolution. Based on this, a Markov transition matrix is constructed, which is then extended to a Markov Transition Field to achieve image encoding [37]
Motif Difference Field (MDF)	The basic idea of a Motif Difference Field (MDF) is to give a time series, set the number of images to be obtained, and then set different step sizes and length time windows to extract a certain segment of the original shape of the time series multiple times, and then obtain the image through combination transformation [40].
Relative Position Matrix (RPM)	The Relative Position Matrix (RPM) contains redundant features of the original time series, making it easier to capture inter-class and intra-class similarity information in the transformed image [35].
Mel spectrogram	Mel spectrogram is a spectrum representation method widely used in speech processing and audio analysis, which converts frequencies into scales closer to human ear perception based on Mel scales. Mel spectrograms can effectively capture speech features and tone information in audio signals [16].

**Table 3:** Dataset-specific information.

Category	Number of Nor	Number of Out	Number of In	Number of All	Size
Image dataset	1059	1059	1059	1059	224 × 224

**Table 4:** Comparison of different backbone networks in the preliminary experiment.

Network Model	Test Accuracy (%)	Training Time (s)
EfficientNet	98.47	7140.93
EfficientNetv2	98.87	8967.32
MobileNet	97.83	6834.08
RegNet	99.12	9050.51
SqueezeNet	93.49	6549.47

**Table 5:** Computer hardware environment parameters.

Hardware	Parameter Configuration
CPU	AMD Ryzen 7 5800H with Radeon Graphics 3.20 GHz
GPU	NVIDIA GeForce RTX 3060 Laptop GPU
Memory	16.0 GB

**Table 6:** Computer software environment parameters.

Software	Configuration
Operating system	Windows 11
Development platform	MATLAB R2023a and PyCharm Community Edition 2022.3.2
Development framework	PyTorch 2.1

**Table 7:** Results.

Imaging Dataset	Test Set Accuracy (%)	Training Time (s)
GADF	99.52	10,261.50
GASF	95.98	10,051.75
STFT	99.68	10,080.96
MTF	97.27	10,234.63
MDF	99.20	10,115.19
RPM	98.63	10,361.98
Mel spectrogram	99.60	10,017.12

**Table 8:** Mathematical definitions of the five image-level feature fusion strategies.

Fusion Strategy	Mathematical Definition
Weighted summation	$X_{f}=\sum_{k=1}^{K}w_{k}X_{k},w_{k}=\frac{\mathrm{Ac}c_{k}}{\sum_{j=1}^{K}\mathrm{Ac}c_{j}}$
Feature summation	$X_{f}=\sum_{k=1}^{K}X_{k}$
Feature averaging	$X_{f}=\frac{1}{K}\sum_{k=1}^{K}X_{k}$
Feature multiplication	$X_{f}=\prod_{k=1}^{K}X_{k}$
Maximum pooling	*X_f_*(*i*,*j*,*c*) = max_1≤*k*≤*K*_*X_k_*(*i*,*j*,*c*)

**Table 9:** Fusion feature datasets (two sets).

Fused Single Features	Feature Fusion Method	Number of Nor	Number of Out	Number of In	Number of All	Size
STFT; Mel spectrogram	Feature weighted summation	1059	1059	1059	1059	224 × 224
STFT; Mel spectrogram	Feature summation	1059	1059	1059	1059	224 × 224
STFT; Mel spectrogram	Feature averaging	1059	1059	1059	1059	224 × 224
STFT; Mel spectrogram	Feature multiplication	1059	1059	1059	1059	224 × 224
STFT; Mel spectrogram	Feature maximum pooling	1059	1059	1059	1059	224 × 224

**Table 10:** Fusion feature datasets (three sets).

Fused Single Features	Feature Fusion Method	Number of Nor	Number of Out	Number of In	Number of All	Size
STFT; Mel spectrogram; GADF	Feature weighted summation	1059	1059	1059	1059	224 × 224
STFT; Mel spectrogram; GADF	Feature summation	1059	1059	1059	1059	224 × 224
STFT; Mel spectrogram; GADF	Feature averaging	1059	1059	1059	1059	224 × 224
STFT; Melspectrogram; GADF	Feature multiplication	1059	1059	1059	1059	224 × 224
STFT; Mel spectrogram; GADF	Feature maximum pooling	1059	1059	1059	1059	224 × 224

**Table 11:** Fusion feature datasets (four sets).

Fused Single Features	Feature Fusion Method	Number of Nor	Number of Out	Number of In	Number of All	Size
STFT; Mel spectrogram; GADF; MDF	Feature weighted summation	1059	1059	1059	1059	224 × 224
STFT; Mel spectrogram; GADF; MDF	Feature summation	1059	1059	1059	1059	224 × 224
STFT; Mel spectrogram; GADF; MDF	Feature averaging	1059	1059	1059	1059	224 × 224
STFT; Mel spectrogram; GADF; MDF	Feature multiplication	1059	1059	1059	1059	224 × 224
STFT; Mel spectrogram; GADF; MDF	Feature maximum pooling	1059	1059	1059	1059	224 × 224

**Table 12:** Results of two sets.

Feature Fusion Method	Test Set Accuracy (%)	Training Time (s)
Feature weighted summation	97.11	10,018.10
Feature summation	100.00	10,186.37
Feature averaging	99.04	10,118.45
Feature multiplication	94.29	10,421.76
Feature maximum pooling	97.91	10,177.33

**Table 13:** Results of three sets.

Feature Fusion Method	Test Set Accuracy (%)	Training Time (s)
Feature weighted summation	99.60	10,062.06
Feature summation	99.84	10,289.77
Feature averaging	98.23	10,076.79
Feature multiplication	83.60	10,479.55
Feature maximum pooling	96.78	10,115.93

**Table 14:** Results of four sets.

Feature Fusion Method	Test Set Accuracy (%)	Training Time (s)
Feature weighted summation	99.52	10,382.37
Feature summation	99.68	10,440.42
Feature averaging	97.99	10,054.46
Feature multiplication	81.35	10,611.65
Feature maximum pooling	100.00	10,110.45

## Data Availability

The experimental datasets used in this study are available from the corresponding author upon reasonable request. Since the related research is still ongoing, the source code is not publicly available at this stage. Requests for the source scripts used for data segmentation, time-series imaging, feature fusion, and model training will be carefully considered by the authors according to the purpose and reasonableness of the request. The manuscript provides detailed descriptions of the data segmentation strategy, time-series imaging procedure, feature fusion operations, backbone network comparison, and model training settings to support reproducibility.
